# Differences in characteristic morphological variability among distal femurs based on sex and ethnicity

**DOI:** 10.1302/2633-1462.73.BJO-2025-0380.R1

**Published:** 2026-03-23

**Authors:** Nadim Ammoury, Michael J. Dunbar, Jerry D'Alessio, Janie Astephen Wilson

**Affiliations:** 1 Dalhousie University, Halifax, Canada; 2 Nova Scotia Health, Halifax, Canada; 3 Stryker, Mahwah, New Jersey, USA

**Keywords:** Knee Joint, Morphology, 3D model, Arthroplasty, Robotic surgery, Personalization, distal femoral, femora, intercondylar notch, surgical approaches, joint mechanics, CT scans, variance, medial condyles, anterior distal femora, knee arthroplasty implant

## Abstract

**Aims:**

The morphology of the distal femur varies widely among individuals and has a direct impact on knee joint mechanics and function. Understanding this variability is essential in improving surgical planning and implant design, especially as current tools and approaches move toward more anatomically informed and personalized technique. This study sought to characterize the major modes of morphological variability in the adult human distal femur and to examine morphometric differences based on sex and ethnicity.

**Methods:**

A dataset of 1,686 distal femurs from a CT scan-based database was analyzed. A total of 15 morphometric dimensional and angular variables were assessed, and principal component analysis (PCA) was employed to identify key modes of variability. Morphological differences were examined between male and female femurs and among self-identified Caucasian and Asian ethnic groups.

**Results:**

Five principal components (PCs) explained over 90% of the total variance in the original morphometric variables. Male and Caucasian femurs were significantly larger than female and Asian femora respectively (PC1; 58.3% variability explained). There were characteristic variations in the trochlear anatomy, with female and Asian femurs exhibiting more elevated anterior distal femora (PC2; 13.6%). Variability in the intercondylar notch (PC3 11%) and femoral aspect ratio (PC5; 4.9%) were sex-specific, with female femora having relatively less anteriorly elevated medial condyles, larger AP height, and relatively narrower in the AP direction than male femora. However, variability in the condylar twist angles (PC4; 6.2%) was not different based on sex or ethnicity.

**Conclusion:**

This study characterized morphological variability in a large sample of distal femora, with key differences noted based on sex and ethnicity. The results support further consideration of this variability in knee arthroplasty implant design options and surgical approaches.

Cite this article: *Bone Jt Open* 2026;7(3):407–416.

## Introduction

The morphology of the distal femur varies widely among individuals and is influenced by factors such as sex,^1,2^ ethnicity^1,3^ and morphotypes.^2^ These morphological differences play a critical role in knee joint biomechanics^4^ and have been implicated in several clinical conditions. Abnormal shape features may contribute to the progression of knee osteoarthritis (OA).^5,6^ Moreover, bone shape has been shown to influence surgical outcomes. Distal femur morphology is associated with increased rotatory instability following ACL rupture and reconstruction,^7^ and patellar/ trochlear morphology impacts knee function and anterior knee pain after unicompartmental knee arthroplasty.^8^ Understanding distal femoral morphology is therefore essential to better understanding disease onset and progression, as well as for improving surgical planning and outcomes.

Recent advancements in surgical technologies, such as computer-assisted surgery robotic-assisted knee arthroplasty, which have been shown to improve the precision and accuracy of implant placement and alignment,^9^ have made it increasingly feasible to account for individual anatomy and bone morphology during surgery. However, most knee implant designs and surgical planning tools often overlook population variability, making it difficult to achieve adequate morphometric match for many patients.^10^ Mahfouz et al^1^ identified sex and ethnic differences in the morphology of the distal femur and proximal tibia using a bone atlas of 1,000 healthy adult knees, while Koh et al^11^ identified mediolateral tibial plateau shape differences in a Korean population sample, highlighting the limitations of one-size-fit-all implant designs.^3^

Many studies that have investigated knee morphology have used 2D radiological images,^2,7,12^ potentially excluding relevant morphologic features, and suffering from errors associated with misalignment of the images with anatomical axes.^1^ Moreover, the common approach of analyzing individual dimensional variables may fail to capture the correlation structure among morphometric variables, making it difficult to synthesize and interpret these findings together to inform surgical approaches. Data reduction techniques such as principal component analysis (PCA)^13^ can exploit the correlation structure among variables and define new features that maximally explain uncorrelated directions of variability. PCA has been used to study morphological variability of the tibia and femur,^1^ but only to normalize observations to the direction of highest variability.

The primary objective of this study was to objectively characterize the major, uncorrelated directions of variability in morphology of the adult human distal femur, and to examine differences in the distal femur morphology between male and female and Caucasian and Asian distal femora.

## Methods

### Data

Femoral data were obtained from the SOMA database (SOMA; Stryker, USA), which comprises over 15,000 deidentified whole-bone CT scans and associated 3D bone models collected for research and development purposes.^14^ The database includes bones from across the entire human skeleton which were obtained from patients imaged for non-orthopaedic medical indications. From this database, a total of 1,686 femora with no evidence of degenerative arthritis were included in this analysis. Data collection and deidentification were performed under institutional and regulatory approval by the database holder, with the investigators (JAW, JDA) accessing only fully anonymized data. Demographics are included in Table I.

**Table I.** Patient demographics based on sex and ethnicity.

Variable	n	Mean age, yrs (SD; range)	Mean height, cm (SD; range)	Mean mass, kg (SD; range)	Mean BMI kg/m^2^, (SD; range)
All participants	1,686	61.7 (16; 19 to 109)	170 (9.3; 142 to 199)	76.3 (16.4; 37 to 161)	26.4 (5.3; 14.3 to 54.7)
Male femora	924	61.4 (15.3; 19 to 109)	174.9 (7.7; 154 to 199)	80.2 (15.4; 37 to 161)	26.1 (4.5; 14.3 to 50.8)
Female femora	760	61.9 (16.7; 19 to 93)	164.6 (7.6; 142 to 192)	72.0 (16.4; 42 to 145)	26.6 (6.0; 15.2 to 54.7)
Caucasian femora	1,242	63.5 (15.0; 19 to 93)	170.2 (9.3; 142 to 199)	76.2 (16.3; 37 to 145)	26.3 (5.2; 14.3 to 54.7)
Asian femora	193	49.8 (17.3; 19 to 86)	167.2 (5.8; 161 to 180)	71.5 (11.8; 57 to 90)	25.4 (2.8; 22 to 29.4)

**Table II.** Description of original 15 morphologic distal femur variables included in the analysis, based on the SOMA sample included in this study.

Variable	Description	Mean (SD)	Min to max
V1, AP SIZE (mm)	Minimum distance between AP sizing point and posterior plane, where sizing point is the most proximal portion of distal anterior intercondylar groove with negative curvature (see Figure 1, left panel)	59.2 (4.3)	45.8 to 73.2
V2, OVERALL AP (mm)	Minimum distance between prominence of lateral anterior condyle and posterior plane.	66.4 (4.6)	53.6 to 80.4
V3, LZX (mm)	Elevation of lateral anterior condyle relative to AP sizing point in direction normal to posterior plane	22.4 (15.8)	0.3 to 47.1
V4, MZX (mm)	Elevation of medial anterior condyle relative to AP sizing point in direction normal to posterior plane	19.7 (15.7)	0.1 to 43.9
V5, VZX (mm)	Elevation of anterior portion of intercondylar groove relative to AP sizing point in direction normal to posterior plane	16.6 (15.5)	0 to 38.8
V6, AP ANGLE (°)	Angle of AML vector relative to posterior plane	4.6 (2.8)	-10.2 to 12.3
V7, TEA (mm)	Interepicondylar width	84.6 (6.6)	68.2 to 101.9
V8, CAP (mm)	Posterior aspect of intercondylar notch to most anterior portion of intercondylar groove, in direction perpendicular to both TEA and distal anatomical axis	40.1 (3.4)	29.5 to 50.7
V9, MAP (mm)	Medial anterior-posterior height (‘depth’ of medial condyle); connects pair of medial vertices of AML and PML	65.9 (4.8)	52.4 to 80.2
V10, LAP (mm)	Lateral anterior-posterior height (‘depth’ of lateral condyle); connects pair of lateral vertices of AML and PML	66.4 (4.6)	53.9 to 80.4
V11, AML (mm)	Anterior medial-lateral axis, Connects medial and lateral anterior points (trochlear linear)	35.2 (3.6)	25.8 to 46.2
V12, PML (mm)	Posterior medial-lateral axis, Connects medial and lateral posterior points (posterior condyle line)	53.0 (4.2)	41.9 to 64.1
V13, DML (mm)	Distance between most distal aspects of medial and lateral condyles	51.4 (4.2)	40.4 to 62.3
V14, RML (mm)	Resected width, the widest pair of points on a plane in the TEA direction. (standard distal resection plane perpendicular to mechanical axis and 10 mm proximal)	73.8 (6.0)	58.5 to 90.6
V15, CTA (°)	Condylar twist angle, angle between TEA and posterior condylar axis	6.8 (1.6)	1 to 13.3

AML, anterior mediolateral distance; AP, anteroposterior; CAP, intercondylar groove anteroposterior distance; CTA, condylar twist angle; DML, distal mediolateral distance; LAP, lateral anteroposterior distance; LZX, elevation of lateral anterior condyle; MAP, medial anteroposterior distance; MZX, elevation of medial anterior condyle; PML, posterior mediolateral distance; RML, resected mediolateral distance; TEA, transepicondylar axis; VZX, elevation of anterior portion of intercondylar groove.

**Fig. 1:**
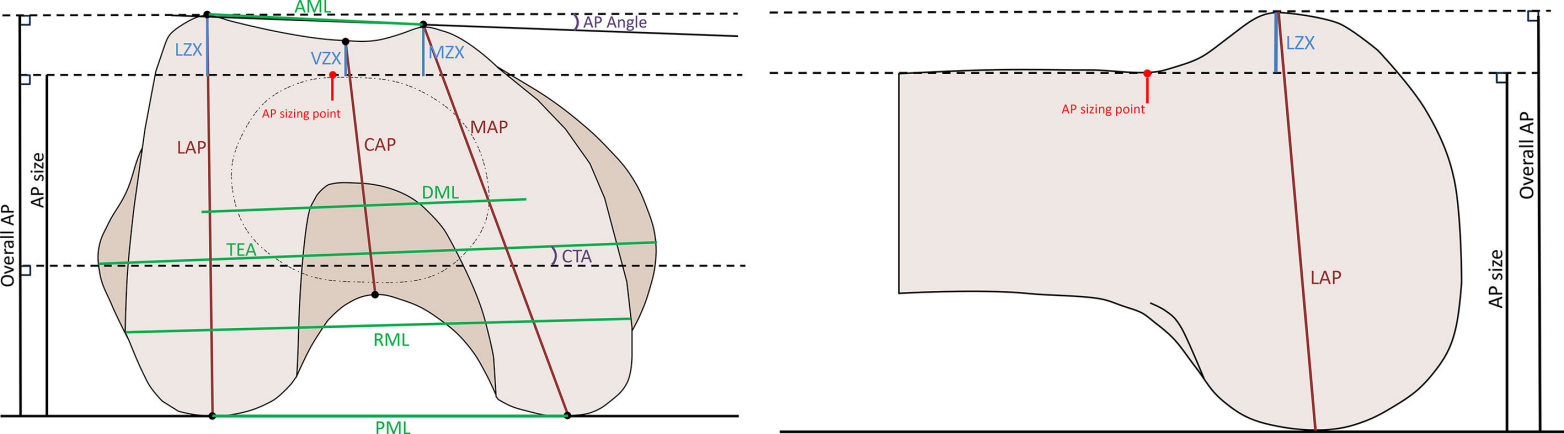
Distal femur measurements. Distal femur measurements viewed from the distal end (left) and lateral end of a right femur (right). The dotted circular line on the distal view represents a transverse projection of the femoral shaft at the anteroposterior sizing point.

In all, 15 dimensional and angular variables were measured from each included distal femur^15^ to characterize distal femur morphology (V1 to V15; Table II, Figure 1),^15^ and were examined for normality and correlation structure. Data was also subdivided into male and female femora, as well as into four self-identified ethnicity categories: Caucasian, African, Asian, and Middle Eastern.

### Principal component analysis model

Principal component analysis (PCA)^13^ is a multivariate statistical analysis technique that transforms a set of correlated variables into a smaller set of uncorrelated components that capture dominant patterns of variability in the data. This method was used to reduce the dimensionality of the data contained in the original 15 variables, and to provide salient features within this set of variables that maximally explain uncorrelated directions or features of variability among the distal femora. Uncorrelated directions of variability among the distal femora were identified using PCA. Principal components (PCs) explaining > 90% of total variance^16^ were retained and interpreted based on coefficient magnitudes and variance contribution of the original variables in each PC. All modelling was performed with custom code in Matlab (MathWorks, USA).

### Data analyses

Student’s two-tailed *t*-tests were used to examine male/female and Caucasian/Asian differences in original variables and PCs (*α* = 0.05). Ethnicity analysis was limited to Caucasian/Asian groups based on insufficient sample sizes of African and Middle Eastern femora for a meaningful characterization.

## Results

### Data analyses

**Fig. 2:**
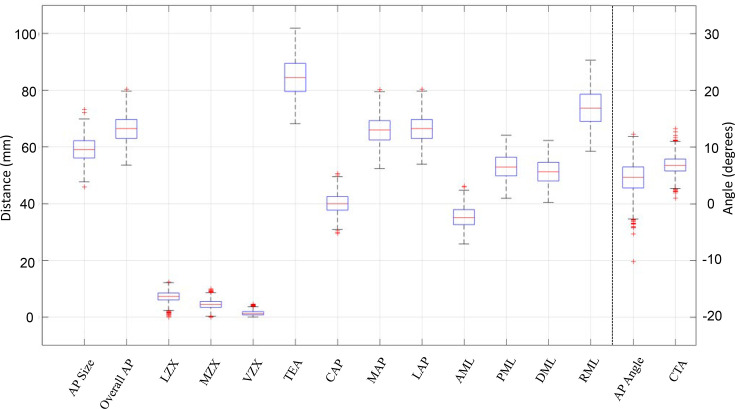
Distribution of original variables. Distribution of the original 15 morphologic distal femur variables included. Median, upper and lower quartiles, whisker (1.5-times the IQR beyond respective quartiles), and data points beyond whiskers are shown.

The original 15 variables were tested for normality and found to be normally distributed (Figure 2). PCs 1 to 5 explained 93.9% of the cumulative variability of the original dataset, and were retained for interpretation and analysis.^16^ The remaining 6.1% variability was explained by PCs 6 to 15, which were not interpreted or included in the current analysis, as our focus was to describe and compare the dominant patterns of morphological variability.

### Interpretation of PC1 through PC5

**Fig. 3:**
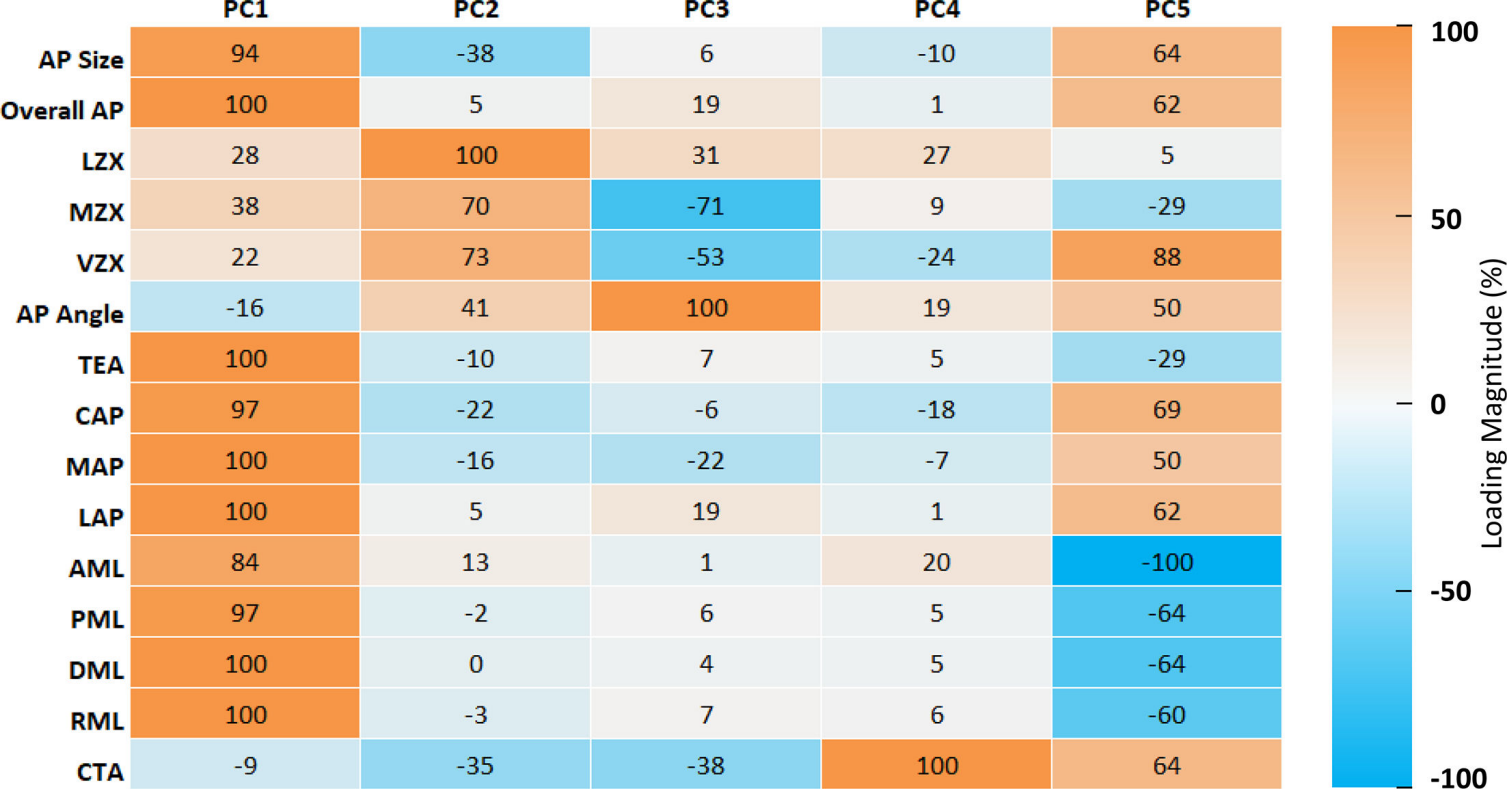
Principal components. Heatmap of the loading vector coefficients of principal components (PCs) 1 to 5, scaled to the largest absolute loading in each PC. The loading magnitudes represent the coefficients of the original variables in each PC, and therefore the relative contribution of each original variable to the variability pattern described by a given PC. Strong positive and negative contributions are shown in orange and blue, while low contributions (near 0) are in grey.

**Fig. 4:**
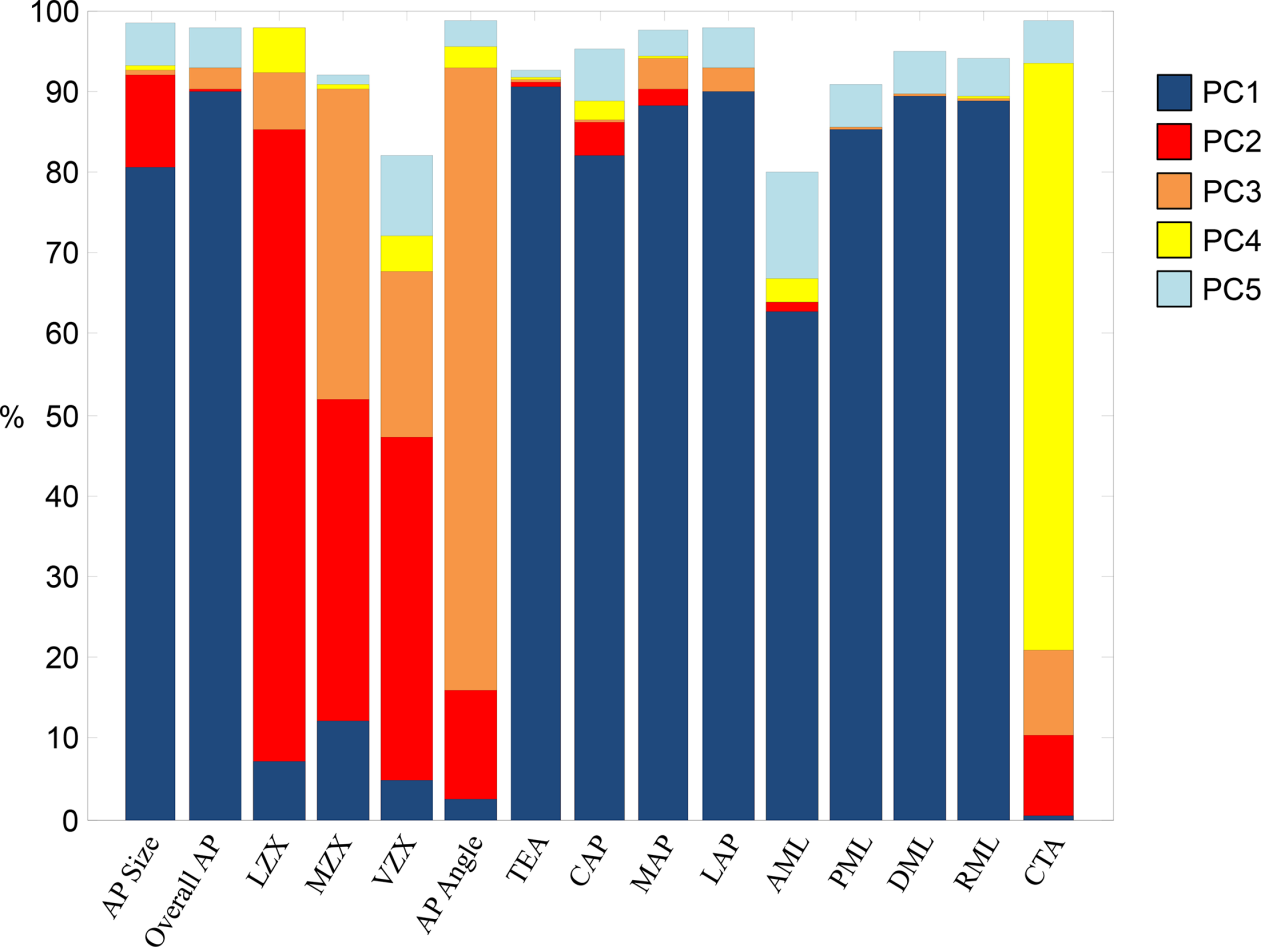
Cumulative percent variation explained of principal components 1 to 5. The graph shows, for each original variable, the cumulative proportion of its total variance explained by the first five principal components (PCs). Each coloured segment within a bar represents the contribution of an individual PC to that variable’s variance, allowing visualization of which components capture most of the variability of a given variable. Variables whose cumulative variation reaches values close to 100% are largely captured by PCs 1 to 5, while those with lower cumulative percentages have residual variability explained by subsequent components not included in this analysis.

**Fig. 5:**
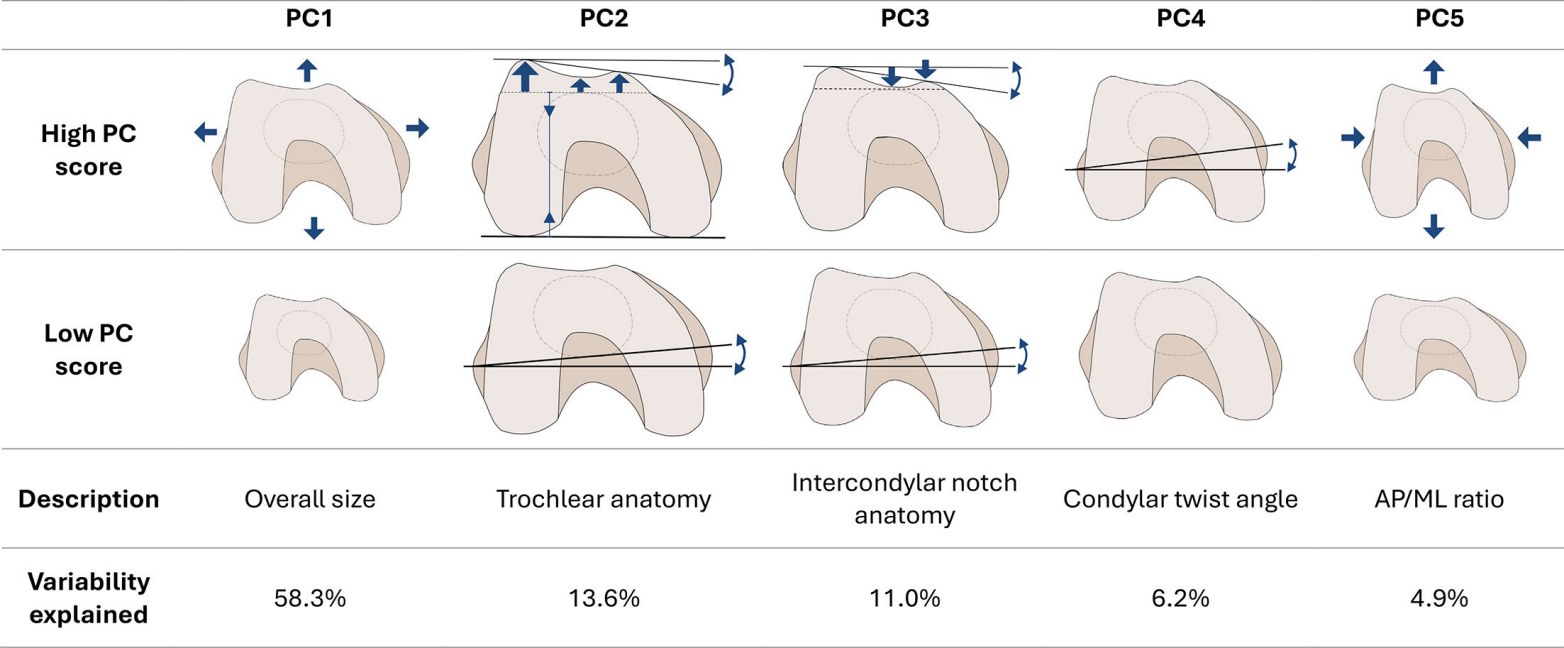
Representation of high and low scores for principal components (PCs) 1 to 5. Arrows indicate contributions of the morphology dimensions. A right femur is shown, i.e. lateral and medial condyles are on the left and right-hand side respectively. Interpretation and variability explained by each PC are also shown.

Figure 3 shows PC loading vector coefficients for PC1 through PC5. Figure 4 shows the relative added contribution of each PC to the variability in the original 15 variables. Figure 5 offers a visual representation of the contributions of distal femur morphology dimensions to each PC.

### PC1: Distal femur size

PC1 explained most of the variability (58.3%) in the original 15 variables. PC1 captured an overall size feature of the distal femur, with equal and positive weights from several size dimensions. A high PC1 score represented a distal femur with relatively large AP and ML dimensions.

### PC2: Distal femur shape 1 – trochlear anatomy

PC2 (13.6% variability) described a difference between condylar elevation measures and AP angle relative to AP size and CTA, with high PC2 scores associated with larger LZX, VZX, and MZX and AP angle, relative to smaller AP size and CTA.

### PC3: Distal femur shape 2 – intercondylar notch anatomy

PC3 (11% variability) characterized the relative size of the CTA, medial condyle and intercondylar elevation, relative to the AP angle. A high PC3 score represented a relatively large AP angle compared to relatively small CTA, and MZX and VZX distances.

### PC4: Distal femur condylar twist

PC4 (6.2% variability) was defined almost solely by the contribution of the CTA. A high PC4 score represented a relatively large CTA.

### PC5: Distal femur shape 3 – AP/ML ratio

PC5 (4.9% variability) captured the relative difference between the anterior-posterior and medial-lateral dimensions of the distal femur. A high PC5 score represented a ‘more narrow’ distal femur, meaning larger AP size, Overall AP, VZX, AP Angle, CAP, MAP and LAP, relative to a smaller AML, PML, DML, and RML.

### Demographic-specific results

Sex-specific differences in distal femur morphology: Statistically significant (p < 0.010) differences between morphological measurements of male and female distal femora were found in all original variables, except VZX, the intercondylar groove dimension (Table III), with female femora being relatively smaller and more angular.

**Table III.** Original variable descriptive statistics by sex and ethnicity: descriptive statistics of original 15 variables for 925 male and 760 female femora, and for 1,242 Caucasian and 193 Asian femora.

Variable	Mean male (SD)	Mean female (SD)	Mean Caucasian (SD)	Mean Asian (SD)
V1, AP SIZE (mm)	61.7 (3.3)	56.0 (3.1)*	59.4 (437)	57.8 (4.3)*
V2, OVERALL AP (mm)	69.2 (3.5)	63.0 (3.3)*	66.6 (4.5)	64.4 (4.5)*
V3, LZX (mm)	7.44 (1.9)	6.93 (1.8)*	7.23 (1.9)	6.63 (1.7)*
V4, MZX (mm)	4.67 (1.6)	4.15 (1.6)*	4.47 (1.6)	3.79 (1.4)*
V5, VZX (mm)	1.35 (0.9)	1.36 (0.9)	1.37 (0.9)	1.24 (0.7)
V6, AP ANGLE (°)	4.29 (2.7)	4.92 (2.9)*	4.54 (2.8)	4.80 (2.5)
V7, TEA (mm)	89.2 (4.5)	79.0 (3.8)*	84.8 (6.5)	82.3 (6.2)*
V8, CAP (mm)	42.0 (2.7)	37.8 (2.6)*	40.4 (3.4)	38.5 (3.3)*
V9, MAP (mm)	68.8 (3.7)	62.4 (3.5)*	66.2 (4.7)	63.8 (4.7)*
V10, LAP (mm)	69.2 (3.5)	63.0 (3.3)*	66.6 (4.5)	64.5 (4.5)*
V11, AML (mm)	37.3 (2.7)	32.6 (2.7)*	35.2 (3.6)	34.4 (3.4)†
V12, PML (mm)	55.9 (2.9)	49.5 (2.6)*	53.4 (4.1)	50.5 (3.9)*
V13, DML (mm)	54.3 (2.8)	47.8 (2.5)*	51.6 (4.1)	49.4 (4.0)*
V14, RML (mm)	78.1 (4.0)	68.7 (3.4)*	74.1 (6.0)	71.4 (5.5)*
V15, CTA (°)	6.71 (1.5)	6.93 (1.6)†	6.84 (1.5)	6.88 (1.7)

The p-value represents result of testing null hypothesis that male and female, or Asian and Caucasian femur variable means are equal against alternative hypothesis that they are not (Student’s two-tailed *t*-test).

* p-value < 0.0001.

† p-value < 0.01.

Differences in characteristic morphometric features (PCs) were also identified between male and female distal femora in PC1 (p < 0.001), PC2 (p < 0.001), PC3 (p = 0.004), and PC5 (p < 0.001). Female distal femora were smaller overall than male femora (PC1). Female distal femora had larger PC2 scores than male, indicative of an anterior condylar shape difference characterised by relatively larger LZX, MZX, VZX, and AP angle, compared to relatively smaller AP size and CTA angle. Female femora had larger PC3 scores than male, and therefore relatively smaller CTA, and MZX and VZX compared with relatively larger AP angles. Female femora also had higher PC5 scores, or more narrow distal femora.

Ethnic differences in distal femur morphology: Out of the 1,686 patients, 1,242 identified as Caucasian, 193 as Asian, 51 as African, and 19 as Middle-Eastern, with 181 patients not indicating their ethnicity. Differences in original dimensional variables between Caucasian and Asian distal femora were found in all dimensions other than VZX, the intercondylar groove dimension, and the angular measurements (AP angle and CTA; Table III). Asian distal femora were characterized by smaller dimensions (V1, V2, V3, V4, V7, V8, V9, V10, V11, V12, V13, and V14) than Caucasian distal femora.

Differences in morphometric features (PCs) between Caucasian and Asian distal femora were identified only in PC1 and PC2. Asian femora were overall smaller (PC1) than Caucasian femora. Asian distal femora were also characterised by a different anterior condylar shape than Caucasian femora (PC2), with relatively larger LZX, MZX, VZX, and AP angle, compared to relatively smaller AP size and CTA angle.

## Discussion

This study identified five key principal directions of morphological variability in a diverse sample of adult distal femora that cumulatively explained the majority of the variability (> 90%). The first PC explained 58.3% of the variability and captured an overall size feature of the distal femur. This was expected as the first PC typically captures an overall signal amplitude, which in morphology would equate to overall femur size.^1,13^ As hypothesized and consistent with previous literature, female^1,17,18^ and Asian^1^ distal femora were overall smaller (PC1) than male and Caucasian femora. The size range of available off-the-shelf implants are generally not sufficient enough to accommodate smaller femora, common in female^19^ and Asian populations,^20^ which can lead to implant overhang and worse postoperative outcomes.^19^

The second and third PCs both described variability in the shape of the anterior distal femur. PC2 captured a shape feature of the trochlear groove of the distal femur describing relatively large elevations of the anterior aspect of the distal femur and a more angular shape in the AP direction, relative to small anteroposterior size and condylar twist angle. PC3 characterized distal femora with small intercondylar notch and medial condylar elevations compared to relatively large AP angles. Few studies have categorized anterior elevation of femoral condyles, with some associating lower anterior condylar height with trochlear degeneration.^21^ In contrast, the present study captured high variability in anterior condylar shape in a sample with no known pathology, warranting more attention to this aspect of the distal femur in morphometric analyses. Variability in trochlear offset suggests that patients with native anatomy substantially higher or lower than the range accommodated by off-the-shelf implants may experience altered patellofemoral mechanics, potentially leading to a sensation of an unnatural knee and reduced postoperative satisfaction.^22^ Indeed, many tibial implants are externally rotated to accommodate better patella-femoral tracking, but which may result in implant overhang in the tibiofemoral space and compromising tibiofemoral kinematics.^23^ A recent large-scale analysis by Rosa et al^24^ support these findings, showing that over 40% of knees fall outside the design range of currently available trochlear implants. Identifying the continuum of trochlear morphology also provides a reference for identifying dysplastic morphologies, which may influence patellofemoral instability and surgical planning. Similarly, intercondylar notch morphology is relevant for posterior-stabilized implant designs and ligament reconstruction procedures as it affects graft placement and impingement risk.^25^ Medial and lateral anterior tibiofemoral joint asymmetry contributes to joint biomechanics, most notably to the coupled rotational kinematics with knee flexion/extension (i.e. the screw-home mechanism).^26^ Understanding anterior morphological variability and its impact on joint mechanics, both in healthy and pathological knees may help identify specific morphological features that contribute to altered kinematics after arthroplasty. This knowledge can guide personalized surgical approaches, such as implant design modifications or alignment strategies, to address residual mechanical deficits and improve postoperative function.

In this study, PC2 allowed us to quantify variations in anterior femoral morphology, showing that female and Asian distal femora tended to be more elevated and angular anteriorly. This contrasts with previous studies on sex differences in anterior femoral morphology,^18^ possibly because PC2 also reflected smaller anteroposterior height, higher AP angle and less angular condylar twist. These combined shape features may have shifted the sex association such that female femora appeared more elevated within this multidimensional context, even though traditional univariate measures might not show this. We also found sex differences in the intercondylar notch and anterior medial condylar height (PC3), with female distal femora more angular and less elevated anteriorly on the medial side relatively with less femoral angular twist. Compared to previous studies, this PCA combined anterior shape information among other metrics to provide a more complete understanding of sex and ethnic differences of the anterior shape of the distal femoral condyles, as captured by two high-ranking PCs. Furthermore, these results also highlighted the asymmetrical nature of the distal femur shape which was a dominant feature of variability in the population after size, and one that most current implant designs do not account for.^10,11,17^

PC4 captured variability primarily in the CTA, indicating it did not correlate well with other morphometric variables and was a key feature of variability on its own. Despite capturing a sizable amount of variability (6.2%), no sex or ethnicity differences were found in the CTA, differing from past literature showing sex specific differences,^27^ and different than the statistically significant sex effect we found for the original CTA variable. This may reflect contributions from other morphological features to PC4 variability but that are not strongly associated with sex differences. Furthermore, previous studies have shown that current implants do not restore rotational mechanics to the joint in most cases.^28^ Given the morphologic variability explained by PC4 in a large and diverse adult population, and the fact that the CTA has been correlated with the dynamic internal-external rotation of the knee during movement,^4^ this should be an important morphometric consideration during arthroplasty. Furthermore, it seemed important to note that PC2 (trochlear anatomy) was mutually uncorrelated to PC4, a feature dominated by the condylar twist angle. This is particularly relevant for personalized arthroplasty alignment strategies given that PC4 may be an important independent factor in femoral-tibial joint balancing, suggesting that balancing efforts aiming to restore the CTA may have an unpredictable impact on trochlear anatomy reconstruction, a critical consideration for patellar tracking.^29^ As such, it would be important to consider both trochlear anatomy and condylar twist angle during reconstruction to ensure good rotational mechanics and patellar tracking.

PC5 characterized how narrow or wide the distal femora were in the AP relative to ML directions. This is an important variability feature for consideration in arthroplasty design and prescription as narrower femora, more common in females, as shown here, had a greater chance of implant overhang with current implant designs.^17^ This result corroborates past literature showing more narrow distal femora in females,^1,17^ and more frequent, severe overhang in female patients.^30^ While others have also found that Asian femora tended to be narrower than Caucasian femora,^1,18^ our results did not support this, possibly due to differences in the diversity and make-up of the Asian population in our studies, and methodologically how this feature was captured.

Unfortunately, this study lacked sufficient sample sizes for a more diverse and comprehensive analysis of morphometric differences based on ethnicity. Additionally, ethnicity was self-reported and broadly categorized. For example, 'Asian' potentially encompassed a wide range of populations with distinct morphologies, thus limiting the specificity and generalizability of our findings related to ethnic variations. Future efforts are warranted to understand morphometric diversity among arthroplasty candidates and to inform limitations in current approaches and implants for specific subpopulations. While categorizing the population by biological sex and self-identified ethnicity facilitates our understanding of generalized gaps in implant designs and approaches, large morphological variability and overlap exist within these categories. With larger, more diverse datasets, morphometric analytics without regard to subjective categorizations would be most appropriate.

Most off-the-shelf implant components are designed with mechanical alignment (MA) principle in mind, aiming to restore a neutral limb axis rather than to recreate morphology.^31^ When these implants, optimized for MA, are used in procedures aiming to recreate individual anatomy such as kinematic or anatomical alignment, they may lead to suboptimal functional outcomes because the implant geometry was not originally designed to support these techniques.^23,32,33^ Emerging evidence suggests that alignment strategies aiming to restore anatomy (kinematic and anatomical alignments) may lead to improved kinematics and functional outcomes,^34,35^ but their success may depend on how well implant geometry accommodates morphological variability. Indeed, Bonnin et al^36^ demonstrated that personalized alignment strategies outcomes are improved with custom-made implants. This supports the idea that customized implant design and positioning may be most beneficial when paired with personalized alignment approaches, where both implant geometry and alignment are tailored to individual morphology. Therefore, an improved understanding of knee joint morphology and anatomical alignment variability in the context of their impact on joint mechanics across the population is essential not only to guide the design of implant components, but also the development and investigation of personalized alignment approaches that better account for morphometric variability, which can ultimately improve functional outcomes.^37^

This study focused on distal femur morphometric analysis, with an interpretation relative to its implications for knee arthroplasty. However, we did not have the data to fully categorize the articulating knee joint morphology. Future work should further characterize the variability of the proximal tibia, and the interaction of bone morphology with joint alignments to better inform best practices and innovative approaches to arthroplasty and implant design. Moreover, despite the evident connection between joint morphology and biomechanics,^26^ this has received limited attention. In order to maximize the power of robotics and surgical planning for addressing the continued joint functional deficits postoperatively for many patients, there is a need for investigations based on diverse datasets that link morphometric variability with joint mechanics to inform surgical approaches on a personalized level.

We chose to use PCA to objectively characterize and interpret prominent features in femur morphology in a large sample to understand characteristic shape and size differences based on demographics, primarily to highlight opportunities to improve treatment for specific patient groups who often suffer with worse postoperative outcomes. Further morphometric characterizations for use in phenotyping and personalized prescriptions could take advantage of more specialized approaches that do not require the subjective inclusion of specific dimensional variables. For example, statistical shape modelling circumvents the need for manual dimensional measurements,^38^ and can be more readily used in simulating knee joint’s biomechanics relative to different morphology to develop personalization approaches to target deficits in joint biomechanics.^38^ It is also important to note that this work was conducted on a sample with no known pathologies. The progression of osteoarthritis is accompanied with the development of progressive changes in joint structures, which can include voluminous osteophytes that can greatly alter bone morphology, complicating the models used in this study. Future work should include morphological changes due to disease processes and how they interact with general joint shapes when prescribing arthroplasty treatment based on morphology.

In conclusion, to personalize arthroplasty to the individual knee to maximize postoperative outcomes and function, the variability of the morphometric features of the knee joint should be included in pre-surgical planning. Our results highlight characteristic size and shape differences in the human distal femur in a large sample from a diverse population. Differences in characteristic features of distal femur morphology based on sex and Asian/Caucasian ethnicity highlight opportunities for further refinement in the arthroplasty space to better accommodate the diversity among patients.

**Take home message**

- Distal femur morphology varies systematically across sex and ethnicity, highlighting the importance of accounting for patient-specific morphology in the treatment of orthopaedic conditions.

- These findings support the continued development of more personalized solutions to improve clinical outcomes, joint biomechanics, and patient quality of life.

## Data Availability

The datasets generated and analyzed in the current study are not publicly available due to data protection regulations. Access to data is limited to the researchers who have obtained permission for data processing. Further inquiries can be made to the corresponding author.
